# Synergetic Effect of 2-Methacryloyloxyethyl Phosphorylcholine and Mesoporous Bioactive Glass Nanoparticles on Antibacterial and Anti-Demineralisation Properties in Orthodontic Bonding Agents

**DOI:** 10.3390/nano10071282

**Published:** 2020-06-30

**Authors:** Se Young Park, Kyung-Hyeon Yoo, Seog-Young Yoon, Woo-Sung Son, Yong-Il Kim

**Affiliations:** 1 Department of Orthodontics, Dental Research Institute, Pusan National University, Yangsan 50612, Korea; cdent1213@pusan.ac.kr (S.Y.P.); wsson@pusan.ac.kr (W.-S.S.); 2 School of Materials Science and Engineering, Pusan National University, Busan 46241, Korea; seweet07@pusan.ac.kr (K.-H.Y.); syy3@pusan.ac.kr (S.-Y.Y.); 3 Dental and Life Science Institute, Pusan National University, Yangsan 50612, Korea

**Keywords:** mesoporous bioactive glass, 2-methacryloyloxyethyl phosphorylcholine, anti-demineralisation, antibacterial, orthodontic bonding agent

## Abstract

2-methacryloyloxyethyl phosphorylcholine (MPC) is known to have antibacterial and protein-repellent effects, whereas mesoporous bioactive glass nanoparticles (MBN) are known to have remineralisation effects. We evaluated the antibacterial and remineralisation effects of mixing MPC and MBN at various ratios with orthodontic bonding agents. MPC and MBN were mixed in the following weight percentages in CharmFil-Flow (CF): CF, 3% MPC, 5% MPC, 3% MPC + 3% MBN, and 3% MPC + 5% MBN. As the content of MPC and MBN increased, the mechanical properties of the resin decreased. At 5% MPC, the mechanical properties decreased significantly with respect to CF (shear bond strength), gelation of MPC occurred, and no significant difference was observed in terms of protein adsorption compared to the control group. Composition 3% MPC + 5% MBN exhibited the lowest protein adsorption because the proportion of hydrophobic resin composite decreased; CF (91.8 ± 4.8 μg/mL), 3% MPC (73.9 ± 2.6 μg/mL), 3% MPC + 3% MBN (69.4 ± 3.6 μg/mL), and 3% MPC + 5% MBN (55.9 ± 1.6 μg/mL). In experiments against *S. mutans* and *E. coli*, addition of MPC and MBN resulted in significant antibacterial effects. In another experiment, the anti-demineralisation effect was improved when MPC was added, and when MBN was additionally added, it resulted in a synergetic effect. When MPC and MBN were added at an appropriate ratio to the orthodontic bonding agents, the protein-repellent, antibacterial, and anti-demineralisation effects were improved. This combination could thus be an alternative way of treating white spot lesions.

## 1. Introduction

Enamel demineralisation, also referred to as white spot lesions (WSL), is the most frequent complication associated with fixed orthodontic treatments [1]. The prevalence of WSL during fixed orthodontic treatment ranges widely, from 2% to 96%, with individual teeth with fixed appliances exhibiting a higher prevalence of WSL than on the teeth in the control group [2,3,4,5]. The occurrence of WSL after orthodontic treatment is mainly due to plaque deposits. Fixed orthodontic appliances create an environment for plaque to build up and make it difficult to clean the teeth. In addition, the irregular surfaces of brackets, bands, and wires limit the self-cleansing mechanism by oral muscles and saliva [6]. The deposition of plaque facilitates the attachment of bacteria, and the lower pH due to the metabolism of bacteria leads to WSL [7]. In addition, excessive etching to increase the bonding strength of brackets may increase the susceptibility of enamel surfaces to bacterial action, thereby causing WSL [8].

WSL can be managed via the prevention of demineralisation and promotion of remineralisation of affected lesions, with a variety of methods, e.g., toothpastes, varnishes, mouth rinses, gels, or topical creams containing fluoride, casein phosphopeptide–amorphous calcium phosphate (CPP–ACP), and bioactive glass (BAG), having been reported in literature [9,10]. One of these formulations, BAG, is a bioactive material composed of CaO, Na_2_O, and P_2_O_5_ based on SiO_2_ matrix. BAG releases calcium and phosphorous ions in large amounts and at fast dissolution rates, acting as a source of these ions [11]. A large amount of these ions act as a buffer by raising the pH, thereby preventing demineralisation, and simultaneously, promote the formation of hydroxyapatite (HAp) to induce remineralisation of WSL [12,13,14]. In particular, mesoporous bioactive glass nanoparticles (MBN) not only provide the ability to load other biomolecules but also demonstrate increased bioactivity [15]. Given these advantages, MBN is widely used in the field of biomaterials [16]. 

A zwitterionic material, on the other hand, has the same amount of negatively and positively charged groups on its surface, maintaining overall electrical neutrality [17]. Because of this property, these materials are known to resist nonspecific protein adsorption, bacterial adhesion, and biofilm formation, and recent studies using zwitterionic materials to treat antifouling surfaces have been reported [18,19,20,21]. 2-Methacryloyloxyethyl phosphorylcholine (MPC) is a well-known zwitterionic material. MPC is a methacrylate with a phospholipid polar group in the side chain and is a hydrophilic biopolymer [22]. MPC has a strong protein-repellent action and is grafted to artificial blood vessels, hip joints, and microfluidic devices [23,24].

In the field of dental materials, various attempts have been made to use MPC in protein-repellent dental composites, bonding agents, cements, and coatings to remove bacterial adhesion, reduce acid production, and protect tooth structure [25,26,27,28]. According to Zhang et al., MPC can reduce the adhesion of bacteria but does not have the ability to sterilise, that is, to directly kill bacteria. Furthermore, it does not have any remineralisation capability. Therefore, to effectively inhibit biofilm formation and prevent secondary caries, the addition of antibacterial and remineralisation agents into MPC-containing dental composites is necessary [29]. From that point of view, MPC has been added to fluoride-releasing agents, such as resin-modified glass ionomer (RMGI), light-curable fluoride varnish, and surface pre-reacted glass-ionomer-filled resin-based composite (SPRG-filled RBC), to investigate their respective antibacterial and remineralising effects, wherein significant increases in the effects have been observed [30,31,32]. MBN does not have protein adsorption properties. In addition, the antibacterial effect of MBN is influenced by glass composition, bacterial species, and sample concentration [13]. Using MPC together can complement and elevate these points. Nevertheless, studies on dental composites that contain MBN and MPC are still lacking. Therefore, the objectives of this study were to synthesise biofunctional orthodontic bonding agents via the mixing of MPC, that has antibacterial and protein-repellent capabilities, and MBN, which has remineralisation abilities, at various ratios, and to investigate the mechanical properties and protein adsorption, antibacterial, and anti-demineralisation abilities of the synthesised orthodontic bonding agents.

## 2. Experimental Section

### 2.1. Synthesis of Mesoporous Bioactive Glass Nanoparticles

MBN was synthesised via a modified sol–gel method [33]. Twenty millilitres of ethanol (CH_3_CH_2_OH; Samchun, Pyeongtaek, South Korea), 2 mL of aqueous ammonia (NH_4_OH; Samchun, Pyeongtaek, Korea), 10 mL of 2-ethoxyethanol (C_2_H_5_OCH_2_CH_2_OH; Sigma-Aldrich, St. Louis, MO, USA), 3.12 g of calcium nitrate tetrahydrate (Ca(NO_3_)_2_∙4H_2_O; Sigma-Aldrich, St. Louis, MO, USA), and 1 g of hexadecyltrimethylammonium bromide or cetyl trimethyl ammonium bromide (CTAB, CH_3_(CH_2_)_15_N(Br)(CH_3_)_3_; Sigma-Aldrich, St. Louis, MO, USA) were added to 150 mL of distilled water and stirred at 600 rpm for 30 min at room temperature. Then, 5 mL of tetraethyl orthosilicate (TEOS, Si(OC_2_H_5_)_4_; Sigma-Aldrich, St. Louis, MO, USA) was added to the mixture and stirred for 30 min at room temperature. Next, 0.25 mL of triethyl phosphate (TEP, (C_2_H_5_O)_3_PO; Sigma-Aldrich, St. Louis, MO, USA) was added to the mixture and stirred for another 4 h at room temperature. After the formation of white precipitate, it was washed and dried for 24 h at 60 °C in an oven. Finally, it was heat-treated for 5 h in a 600 °C furnace. 

#### Characterisation of MBN

A field-emission scanning electron microscope (FESEM; SU-70, Hitachi, Tokyo, Japan) was used to observe the surface morphology of the synthesised MBN. Surface area and pore size were measured via the Brunauer-Emmett-Teller (BET) method (BELSORP-max, MicrotracBEL Corp., Osaka, Japan).

### 2.2. Preparation of Orthodontic Bonding Agents Containing MPC and MBN

To prepare orthodontic bonding agents for the experiment, the synthesised MBN and commercially available ready-made MPC (Sigma-Aldrich, St. Louis, MO, USA) were mixed with flowable resin (CharmFil Flow; Denkist, Gunpo, South Korea). The components of CharmFil Flow are listed in Table S1. To prevent polymerisation by light during mixing, CharmFil Flow, MPC, and MBN were added to a 2 mL black e-tube and mixed twice, for 10 s in each round of mixing, using a mixer (TORNADO SHM-ALM00; Shinhung, Seoul, South Korean). Five experimental groups (differentiated by weight ratio %) were tested (Table 1). To evaluate their degree of conversion, microhardness, and biological properties, resin discs were prepared via injection of the mixed paste into circular moulds, each with a diameter of 5 mm and height of 1 mm, which were each then covered with a slide glass on top. The resin discs were then subjected to photopolymerisation (VALO; Ultradent, South Jordan, UT, USA) for 20 s.

### 2.3. Mechanical Properties

#### 2.3.1. Degree of Conversion

**Figure 1:**
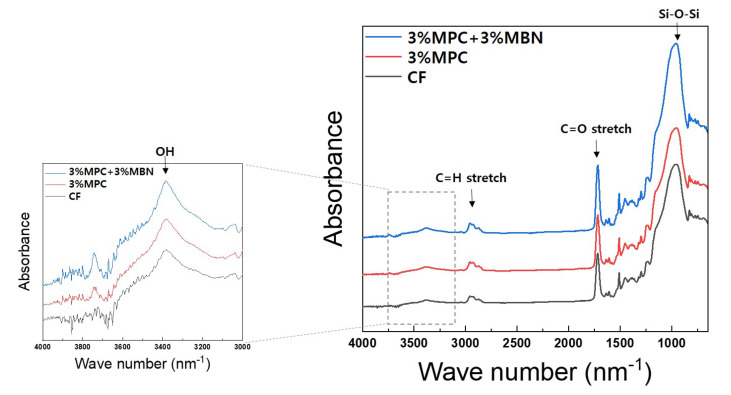
Fourier Transform Infrared Spectroscopy (FT-IR) spectra of CharmFil-Flow (CF), 3% MPC, and 3% MPC + 3% MBN.

The degree of conversion was evaluated using Fourier-transform infrared spectroscopy (FT-IR, Spectrum GX; PerkinElmer, Waltham, MA, USA) and measured via the attenuated total reflectance (ATR) method. FTIR spectrum was recorded for 4000–650 cm^−1^ with 32 scans at a resolution of 4 cm^−1^ (Figure 1). To determine the proportion of double bonds reacted via polymerisation in each group, the absorbance peak areas of methacrylate carbon double bond (aliphatic carbon double bond; peak at 1634 cm^−1^) and internal standard (aromatic carbon double bond; peak at 1608 cm^−1^) before and after polymerisation were measured [34]. The degree of conversion, which was calculated using the following formula, was measured three times per group.

Degree of conversion (%)

(1) $$[1-\{\frac{\left(\frac{aliphatic\;C=C}{aromatic\;C=C}\right)polymer\;before\;conversion}{\left(\frac{aliphatic\;C=C}{aromatic\;C=C}\right)\;polymer\;after\;conversion\;}\}]\times 100$$

#### 2.3.2. Microhardness

To evaluate the mechanical properties of the synthesised orthodontic bonding agents, microhardness was measured via the Vickers test (MVK-H1; Mitutoyo, Kanagawa, Japan). The indentation load was determined via application of a loading of 200 gf to the top surface of the synthesised discs and division of the measured value by the surface area of the indentation. Three samples were used per group, and for each sample, measurements were obtained five times. 

#### 2.3.3. Shear Bond Strength (SBS)

To assess the bond strength of the synthesised orthodontic bonding agents, SBS was measured using a universal testing machine (Instron, Canton, MA, USA). Premolars extracted from orthodontic treatments were used, with 10 premolars used for each group. This study was reviewed and approved by the Institutional Review Board of the Pusan National University Dental Hospital (PNUDH-2019-035). The enamel surfaces to which the brackets were attached were cleaned with pumice without fluorine and washed with water. After being etched for 15 s with 35% phosphoric acid gel (Ultra-Etch; Ultradent, South Jordan, UT, USA), the premolars were then washed with water. The chalky surfaces were checked, after which an adhesive (Scotchbond™ multi-purpose adhesive; 3M, Monrovia, CA, USA) was applied and gently aired for 2 s. The orthodontic bonding agents were applied onto the premolar brackets (Ormco, Orange, CA, USA), which were then placed parallel to the long axis of the teeth. Any additional paste was removed, and the samples were light-cured (VALO; Ultradent, South Jordan, UT, USA) for 5 s at the mesiodistal side of the brackets. The samples were stored in distilled water for 24 h, and SBS was measured with a universal testing machine. The maximum load (N) was measured at a crosshead speed of 1 mm/min, and the bond strength (MPa) was calculated via division of the load by the bracket base area. The bonding failure of the debonded tooth surface was evaluated using the adhesive remnant index (ARI) score, for which the scoring criterion is outlined in Table 2.

### 2.4. Biological Properties

#### 2.4.1. Cell Viability

Cell viability was evaluated using 3-[4,5-dimethylthiazol-2-yl]-2,5-diphenyl tetrazolium bromide (MTT; Sigma-Aldrich, St. Louis, MO, USA) colorimetric assay. Human dental pulp stem cells (hDPSc; Lonza, Alpharetta, GA, USA) were purchased from Lonza (PT-5025) and cultured in Dulbecco’s modified Eagle’s medium (DMEM; Gibco, Grand Island, NY, USA) with 10% foetal bovine serum (FBS; Gibco, Grand Island, NY, USA) and 1% antibiotics (penicillin–streptomycin; Gibco, Grand Island, NY, USA). The hDPSc were seeded in a 96-well plate and incubated for 2 h at 37 °C and 5% CO_2_. The cells were then additionally cultured for 24 h in a continuously diluted extract. Extracts (3 cm^2^/mL) were obtained via dipping of discs sterilised with ethylene oxide (EO) gas in culture media for 24 h and filtering through a 0.2 μm filter. The extracts were diluted with the medium such that the final concentrations were 0%, 12.5%, 25%, 50%, and 100% [35]. Three samples were used per group. Then, 10 μL of MTT solution (5 mg/mL MTT in sterile PBS) was added to each well, followed by incubation for 4 h at 37 °C and 5% CO_2_. Later, the solution was replaced by 100 μL of dimethyl sulfoxide, and the colour was read for 5 min at 37 °C. A microplate reader (Sunrise, TECAN, Männedorf, Switzerland) was used to measure the absorbance at 620 nm.

#### 2.4.2. Protein Adsorption

A protein assay kit (Micro BCA Protein Assay Kit; Thermo Fisher Scientific, Waltham, MA, USA) was used to assess protein adsorption. The discs were sterilised with EO gas, and three samples were used per group. All discs were placed in phosphate buffered saline (PBS; Thermo Fisher Scientific, Waltham, MA, USA) for 1 h at room temperature and then in bovine serum albumin (BSA; Thermo Fisher Scientific, Waltham, MA, USA) for 24 h at 37 °C and 5% CO_2_. The discs were then gently stirred with fresh PBS twice, for 5 min each round of mixing. After the samples were immersed in PBS containing 1% sodium dodecyl sulphate (SDS; Thermo Fisher Scientific, Waltham, MA, USA) for 2 h, the unattached protein was washed with PBS. The BSA concentration of the SDS solution was measured with a protein assay kit. In a 96-well plate, 25 μL of SDS solution was mixed with 200 μL of bicinchoninic acid (BCA) working agent, and the plate was incubated at 60 °C for 30 min. The 96-well plate was then cooled to room temperature, and absorbance at 562 nm was measured using a microplate reader. The standard curve was calculated using the BSA standard, and the amount of protein adsorbed on the surface of the discs was calculated from the protein concentration.

#### 2.4.3. Anti-Bacterial Properties

The anti-bacterial properties were assessed using *Streptococcus mutans* and *Escherichia coli*. The discs were sterilised with EO gas, and three samples were used per group. *S. mutans* cells (strain Ingbritt, ATCC 25157) and *E. coli* cells (strain Ingbritt, ATCC 43888) were cultured in a brain–heart infusion (BHI) broth (BD, Franklin Lakes, USA) at a 37 °C incubator. After incubation, alive *S. mutans* and *E. coli* cells were counted according to an optical dentical density at 650 nm (OD_650_) of 1.0 (equivalent to 2 × 10^8^ colony-forming units). Alive *S. mutans* and *E. coli* cells were harvested by centrifugation (Eppendorf, Hamburg, Germany) at 5000 rpm for 5 min, resuspended in BHI broth at a concentration of 1.0 × 10^5^ CFU/mL for anti-bacterial discs test. After 24 h, the absorbance at was measured with a microplate reader (Tecan, Männedorf, Switzerland) at 650 nm (OD_650_). 

### 2.5. Anti-Demineralisation Properties

#### Anti-Demineralisation Test

The remineralisation effect was evaluated using the pH cycling method devised by Stookey et al. [36]. Premolar teeth extracted from orthodontic treatment and without WSL or enamel defects were used, with five premolars used for each group. The teeth were buried as in the SBS measurement, and transparent tapes were applied, except for the 5 mm × 5 mm area that was to be etched. The tapes were removed after the etching and washing process, and the bracket attachment process was performed in the same way as in the SBS measurement. After the bracket was bonded, the samples were stored in distilled water for 24 h. The cycle of immersion for 6 h in demineralising solution (Biosesang, Seongnam, Korea) and 18 h in remineralising solution (Biosesang, Seongnam, South Korea) was then repeated for 14 d. The compositions of the demineralising and remineralising solutions are outlined in Table S2. Each time the solution was changed, the tooth was immersed in distilled water for 1 min, washed, and dried with gentle air before being added to a new solution. The solution was replaced every week with a freshly prepared one.

**Figure 2:**
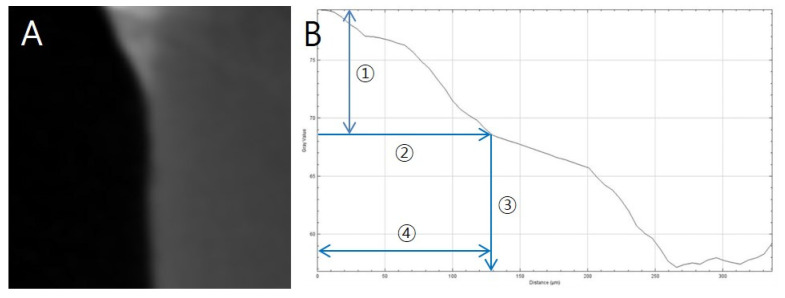
Remineralisation length analysis method. (**A**) Representative CT slice, (**B**) Histogram in ImageJ; ① 87% level of sound enamel brightness; ② Brightness at which enamel loss begins; ③ Depth at which enamel loss begins; ④ Distance from sound enamel to enamel loss (remineralisation length).

After pH cycling, the amount of demineralisation was measured via the method of Paschos et al. [37]. The samples were scanned at 90 kV, 109 μA via micro-computed tomography (micro CT) (inspeXio SMX-90CT Plus Benchtop Microfocus X-ray; Shimadzu, Kyoto, Japan), and analysed using image processing program ImageJ (National Institutes of Health, Bethesda, MD, USA). The lengths of the images were corrected with a scale bar. An area with up to 87% brightness compared to that of a sound enamel was considered as sound enamel, whereas the area with a greater difference in brightness was determined as the area with enamel loss. Remineralisation length was defined as the distance from the end-point where the orthodontic bonding agents were applied, to the point where the sound enamel was located (Figure 2).

### 2.6. Statistical Analysis

All statistical analyses were done with the statistical software SPSS version 21.0 (IBM, Armonk, NY, USA). All data were expressed as mean ± standard deviations and were considered statistically significant when *p*-value < 0.05. The differences between groups were compared using one-way analysis of variance (ANOVA) and with Tukey’s post-hoc test.

## 3. Results

### 3.1. Characteristics of MBN

**Figure 3:**
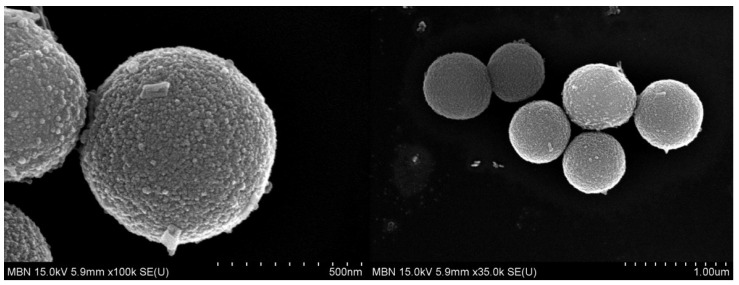
Scanning electron microscope (SEM) image of mesoporous bioactive glass nanoparticle (MBN) powders.

The SEM images confirmed the spherical porous shape of the MBN synthesised via modified sol–gel method (Figure 3).

**Figure 4:**
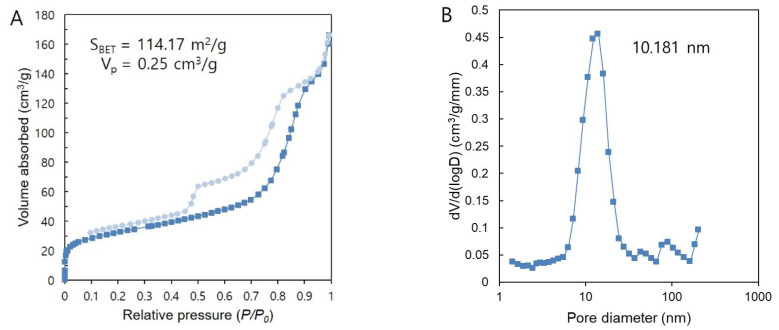
(**A**) N_2_ adsorption–desorption isotherms; (**B**) Pore size distribution of MBN.

The mesoporous structure of MBN was evaluated using N_2_ adsorption–desorption isotherms (Figure 4A). MBN exhibited type IV isotherm characteristics (according to the IUPAC classification) with a type-H1 hysteresis loop, which is a feature of mesoporous materials with relatively uniform pores. The pore sizes of MBN were mostly distributed with an average of 10.181 nm (Figure 4B).

### 3.2. Mechanical Properties

#### 3.2.1. Degree of Conversion

**Figure 5:**
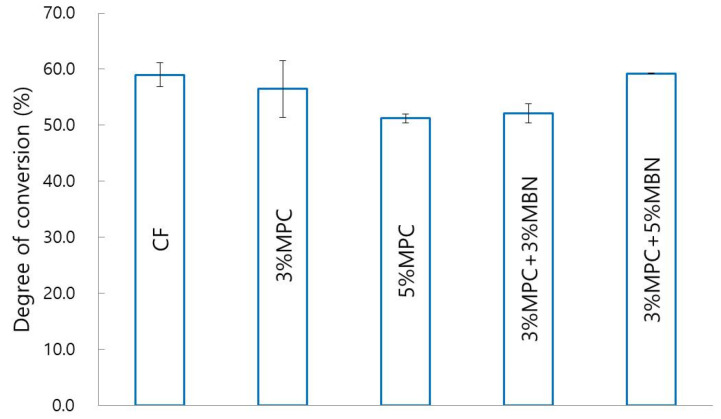
Comparison between degrees of conversion of orthodontic bonding agents containing MPC and MBN. One-way ANOVA was performed (*p* < 0.05). Error bars are shown ± standard error (*n* = 15).

The chemical structures of CF and 3% MPC, 3% MPC + 3% MBN are shown above (Figure 1). For all samples, Si–O–Si and C=O stretching related to CF sample was detected at around 1000 cm^−1^ and 1700 cm^−1^ [38,39]. A peak at around 3390 cm^−1^ was created by OH vibrations. In terms of degree of conversion, there were no statistically significant differences between the groups CF (59.0 ± 2.2%), 3% MPC (56.5 ± 5.1%), 5% MPC (51.3 ± 0.8%), 3% MPC + 3% MBN (52.1 ± 1.7%), and 3% MPC + 5% MBN (59.3 ± 0.1%) (Figure 5).

#### 3.2.2. Microhardness

**Figure 6:**
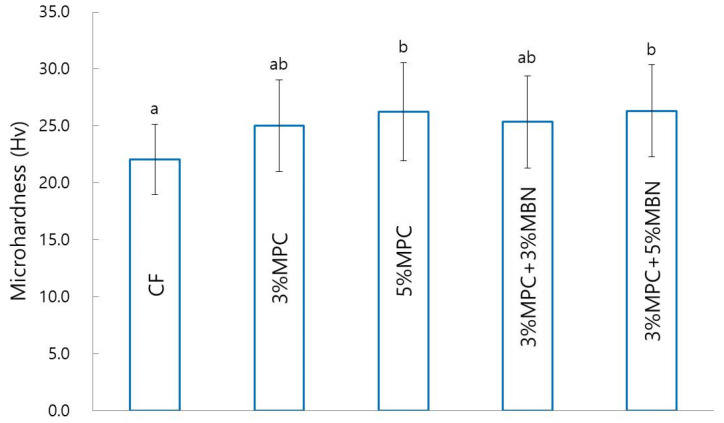
Microhardness comparison of orthodontic bonding agents containing MPC and MBN. Labels with the same letters indicate no statistically significant differences between the groups (*p* > 0.05), as determined via one-way ANOVA. Error bars are shown ± standard error (*n* = 15).

In terms of microhardness, there were significant increases for the 5% MPC (26.3 ± 4.3 Hv) and 3% MPC + 5% MBN (26.3 ± 4.0 Hv) groups, compared to the CF group (*p* < 0.05, Figure 6).

#### 3.2.3. Shear Bond Strength (SBS)

**Figure 7:**
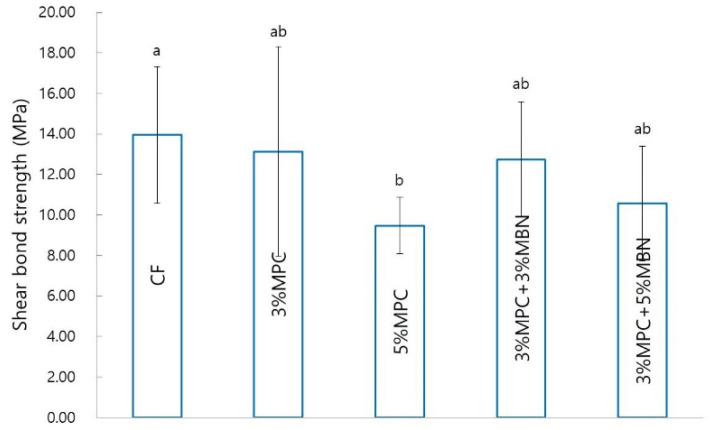
Shear bond strength comparison of orthodontic bonding agents containing MPC and MBN. Labels with the same letters indicate no statistically significant differences between the groups (*p* > 0.05), as determined via one-way ANOVA. Error bars are shown ± standard error (*n* = 10).

The 5% MPC (9.48 ± 1.39 MPa) group exhibited significantly reduced SBS compared to that of the CF (13.95 ± 3.36 MPa) group. The 3% MPC (13.14 ± 5.18 MPa), 3% MPC + 3% MBN (12.74 ± 2.84 MPa), and 3% MPC + 5% MBN (10.57 ± 2.83 MPa) groups demonstrated reduced average values, but these were not significantly different from those of the CF group (Figure 7).

#### 3.2.4. Adhesive Remnant Index (ARI) Score

There were no statistically significant differences in the ARI scores between the groups (Table 3).

### 3.3. Biological Properties

#### 3.3.1. Cytotoxicity Test

**Figure 8:**
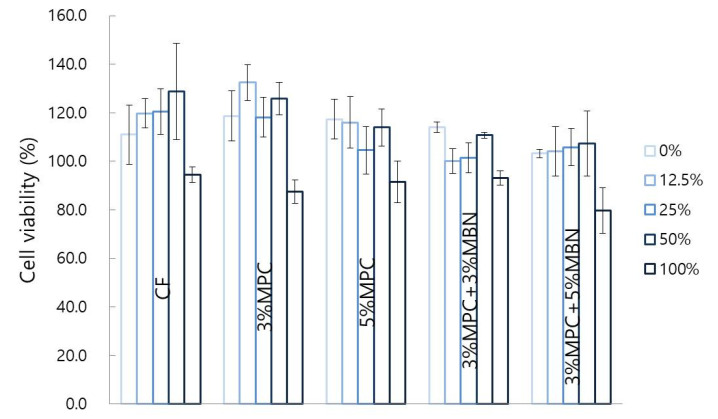
Cytotoxicity test comparison of orthodontic bonding agents containing MPC and MBN. One-way ANOVA was performed (*p* < 0.05). Error bars are shown ± standard error (*n* = 3).

Twenty-four hours after the cell viability test, there were no significant differences in the extract concentrations between the groups (Figure 8).

#### 3.3.2. Protein Adsorption Test

**Figure 9:**
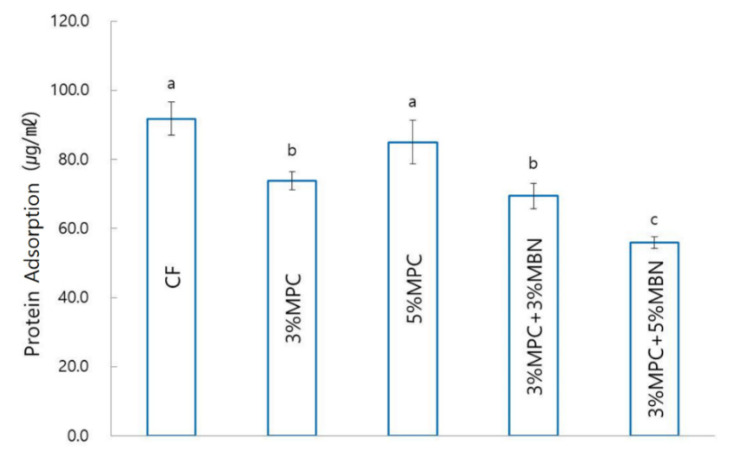
Protein adsorption test comparison of orthodontic bonding agents containing MPC and MBN. Labels with the same letters indicate no statistically significant differences between the groups (*p* > 0.05), as determined via one-way ANOVA. Error bars are shown ± standard error (*n* = 3).

Compared to CF (91.8 ± 4.8 μg/mL), there were significant decreases in protein adsorption for the 3% MPC (73.9 ± 2.6 μg/mL), 3% MPC + 3% MBN (69.4 ± 3.6 μg/mL), and 3% MPC + 5% MBN (55.9 ± 1.6 μg/mL) groups. Of these, the 3% MPC + 5% MBN (55.9 ± 1.6 μg/mL) group had the lowest value (Figure 9). 

#### 3.3.3. Anti-Bacterial Test

**Figure 10:**
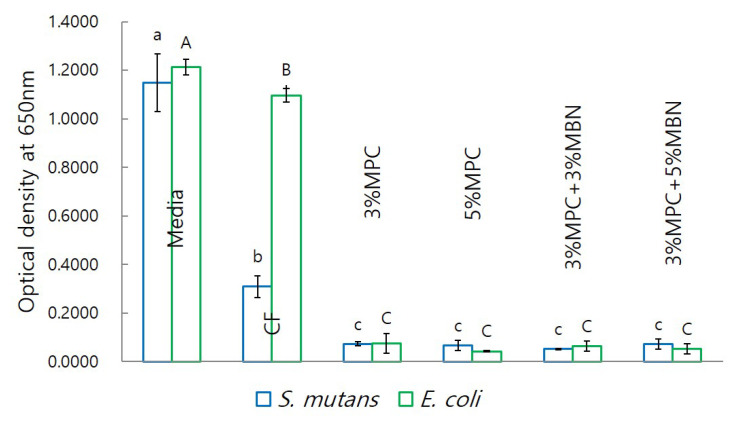
Anti-bacterial test comparison of orthodontic bonding agents containing MPC and MBN. Lower (*S. mutans*) and upper (*E. coli*) case letters are used to distinguish different bacteria group. Labels with the same letters indicate no statistically significant differences between the groups (*p* > 0.05), as determined via one-way ANOVA. Error bars are shown ± standard error (*n* = 3).

In terms of bacterial population, as estimated using optical density, test sample groups exhibited significantly reduced values compared to those for the CF group (Figure 10).

#### 3.3.4. Anti-Demineralisation Test 

**Figure 11:**
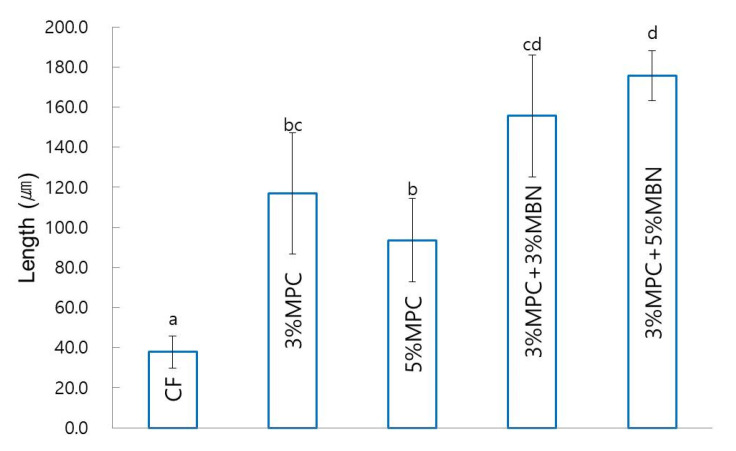
Anti-demineralisation test comparison of orthodontic bonding agents containing MPC and MBN. Labels with the same letters indicate no statistically significant differences between the groups (*p* > 0.05), as determined via one-way ANOVA. Error bars are shown ± standard error (*n* = 5).

In terms of anti-demineralisation capability, the sample groups exhibited a significant increase compared to that of the CF (37.9 ± 8.0 μm) group. The 3% MPC + 5% MBN (175.6 ± 12.5 μm) group had the highest value, followed by the 3% MPC + 3% MBN (155.6 ± 30.5 μm), 3% MPC (116.8 ± 30.2 μm), and 5% MPC (93.6 ± 20.7 μm) groups (Figure 11).

## 4. Discussion

WSL is caused by bacteria that attach to and grow around the bracket, forming a biofilm and producing organic acids [40]. In other words, bacterial attachment in the beginning is an important step in WSL formation, and the initial salivary protein coating is a prerequisite for bacterial attachment [7]. Therefore, it makes sense to develop protein-repellent orthodontic bonding agents, because these agents can inhibit protein adsorption, thereby reducing bacterial adhesion and preventing or minimising WSL. When the pH around the bracket drops to below 5.5, demineralisation becomes prominent and can damage the tooth by dissolving the minerals of the entire enamel; acidic bacteria in biofilm can reduce the pH of the local plaques to 4.5 or even 4 by metabolising carbohydrates [41]. Therefore, to prevent demineralisation of the enamel and WSL, the local pH should be maintained at 5.5 or higher. Education on oral hygiene and the use of fluorine are recommended to prevent the occurrence of WSL, but because these mitigation measures are dependent on patient compliance, these are not effective for children and adolescents [10]. Therefore, various studies have been conducted to reduce the occurrence of WSL through the addition of various substances to the orthodontic bonding agents, rather than through relying solely on patient compliance. 

The purpose of this study was to investigate the mechanical properties, protein adsorption, and antibacterial and anti-demineralisation capabilities of synthesised orthodontic bonding agents that are mixed with MPC and MBN at an appropriate ratio. The study was conducted with four groups using different mass fractions of MPC and MBN: two groups used 3% or 5% MPC, and two groups 3% or 5% MBN with 3% MPC. A previous study found that although the MPC graft polymerisation increased with increasing MPC concentration, the overall polymerisation exhibited gelation at a high MPC concentration, resulting in a decrease in protein-repellent efficiency [42]. According to Zhang et al., the physical properties deteriorate when the proportion of MPC increases above 3% [29,30]. In addition, according to Kwon et al., when the proportion of MPC was 3%, the antibacterial effect was at its highest [31]. For this reason, we wanted to see which group demonstrated the most effective results when MBN is added to 3% MPC. 

An examination of the degree of conversion revealed that while there were no statistically significant differences in the polymerisation rates between groups, the 5% MPC group had the lowest rate. This is consistent with a previous study that when the concentration of MPC exceeds a certain concentration, the overall polymerisation system begins to exhibit gelation and reduced graft efficiency [42]. In the microhardness test, while there were no significant differences between the groups, the CF group had the lowest value, and the microhardness tended to increase as the content of MPC or MBN increased. MPC contains reactive methacrylate groups, which can be copolymerised and covalently bonded to the resin matrix during photopolymerisation. Photo-induced polymerisation can immobilise MPC in the resin matrix through strong covalent bonding, making it durable [42]. Goda et al., showed that the MPC-modified surface layer formed via photo-induced graft polymerisation was resistant to mechanical stress [43]. In conclusion, the hardness of the resin is noted to increase as the content of MPC increases. In the case of MBN, according to Khvostenko et al., composites containing BAG exhibited superior mechanical properties compared to those of a commercially available composite (Heliomolar, Filtek Z250, Filtek Supreme Plus). Moreover, this property was attributed to the fact that BAG acts as a filler and thus has a high filler content and a microstructure morphology that better promoted the toughening mechanisms of crack deflection and bridging [44]. For this reason, as the MBN content increased, the hardness of the resin increased.

SBS and ARI are both important factors related to the bonding of orthodontic appliances. SBS should be high enough for the appliance to avoid failure during treatment, but low enough to allow debonding without damaging the enamel at the end of the treatment [45]. In addition, after debonding, there should be as little residual adhesive on the enamel surface as possible, and the surface should not be damaged. SBS and ARI are related to each other: the higher the SBS, the greater the ARI [45]. In this study, only 5% MPC had a significantly lower SBS value than that of the CF group, whereas there were no significant differences in ARI values between all groups. The 5% MPC group with the lowest SBS exhibited a high ARI value. Furthermore, as the content of MPC or MBN increased, SBS tended to decrease. In this study, as in previous studies, the mechanical properties deteriorated when the mass fraction of MPC was 5% [29,30,31]. In other words, the addition of excessive amounts of MPC or MBN may degrade the mechanical properties of the resin, and in particular, when the MPC is 5% or greater, the mechanical properties undergo a significant decrease. In the cytotoxicity test, the synthesised orthodontic bonding agents showed no clinically significant toxicity compared to the commercially available products (CharmFil-Flow). That is, it can be regarded as clinically usable.

MPC has phospholipid polar groups in its side chain. Phospholipids are a type of lipid in cell membranes and have hydrophilic heads and hydrophobic tails [46]. Once submerged in water, the phospholipids can orient themselves into a bilayer in which the non-polar tails face the inner area of the bilayer, and the polar heads face outward and interact with the water. MPC is protein-repellent, thereby reducing the adhesion of bacteria [23]. Based on these properties, dental composites and bonding agents containing MPC have been studied to reduce bacterial adhesion and acid production and to prevent tooth demineralisation [29,30]. In addition, increasing the mass fraction of MPC in the resin composite increased the amount of MPC, thereby increasing the protein-repellent efficacy of the composite [29]. In this study, when the amount of MPC was increased from 0 to 3%, the amount of protein adsorption significantly decreased, as in previous studies [29,30,31]. In the 5% MPC group, the reduction in protein adsorption was not significant compared to that of the CF group, because of reasons mentioned in similar studies, which reported that the whole polymerisation system exhibited gelation at high MPC concentration, resulting in a decrease in protein-repellent efficiency [42]. Since MBN has a highly hydrophilic property due to the presence of OH groups on the surface, the proportion of hydrophobic resin components in the total proportion decreases as the content of MBN increases [47]. For this reason, the 3% MPC + 5% MBN group exhibited the lowest protein adsorption value. In addition, in this study, the antibacterial ability of the MPC-added group was significantly increased compared to that of the CF group. The result is the same as in previous studies, wherein the biofilm CFU counts of the total microorganisms, total streptococci, and *mutans* streptococci were significantly reduced in the MPC-added group [29,30]. That is, until the start of gelation of MPC, as the concentration of MPC increased, the protein-repellent and antibacterial abilities increased accordingly.

MPC can also enhance the effectiveness of materials with antibacterial effects. Proteins adsorbed from physiological fluid on the resin surface can significantly degrade its antibacterial properties by reducing the contact surface of resin [48]. Indeed, saliva-derived protein films on cationic antibacterial surfaces have been observed to reduce the bactericidal effect [49]. In other words, an MPC-containing composite could likely enhance the antibacterial effects of dental resins by reducing the adsorption of proteins and increasing the contact surface between dental resins and physiological fluids [29]. BAG is a highly reactive substance in aqueous environments, such as the saliva in the oral cavity, and induces the release of calcium and phosphate (PO_4_^3−^) ions from BAG in saliva. When the pH increases because of the released ions, microorganisms can be killed. According to an in vitro study, S53P4, which is a type of BAG, can kill pathogens related to enamel caries (*S. mutans*), root caries (*Actinomyces naeslundii, S. mutans*), and periodontitis (*Actinobacillus actinomycetemcomitans*). S53P4 and high concentrations of BAG compositions in 16 different bacterial cultures exhibited antibacterial properties due to pH increase [13]. Because MPC can improve the effects of materials that have antibacterial effects, addition of MBN may increase the antibacterial effect. When MPC reduces the amount of protein adsorbed on the resin surface, the resin surface becomes more in contact with the physiological fluids. This will increase the reactivity of the BAG in the resin and increase the ion release, thereby increasing the sterilization effect. From all bacteria, just a small fraction exists in the free floating form, while around 99% appear as forming biofilms [50]. Therefore, it can be considered that MPC is effective against most types of bacteria. MBN also shows antibacterial effects on Gram positive bacteria and Gram negative bacteria [13]. In this study, it was confirmed that the antibacterial effect was found in both Gram positive (*S. mutans*) and Gram negative (*E. coli*) bacteria. The MBN-added group exhibited antibacterial effects similar to those of the MPC-only group, mostly likely because the antibacterial effect of MPC itself was very high. To confirm that MPC increases the bactericidal effect of MBN, further research on its bactericidal effect is necessary. 

BAG has been used in numerous studies to remineralise WSL because it can induce apatite formation when it comes into contact with saliva or physiological fluids [51,52]. Because of these advantages, BAG can be used to treat enamel demineralisation, and for this purpose, it is added to toothpaste, preventive gels, and dental materials. BAG can be synthesised in two ways: melting and sol–gel processing. BAG synthesised via the sol–gel method has many advantages because of its fine porous textures and enhanced bioactivity. Furthermore, because the large surface area favours HAp formation, sol–gel-derived BAG exhibits faster HAp formation than that of melt-derived BAG [53]. Vollenweider et al., showed that nanometer-sized particles, compared to micrometer-sized particles, induce faster remineralisation of demineralised teeth [54]. Even in terms of antibacterial effect, nanometric BAG particles exhibit better efficacy because of the high dissolution rate due to an increase in specific surface area [55]. For these reasons, the MBNs in this study were synthesised via the sol–gel method, and validated via SEM and BET analysis.

According to Zhang et al., because MPC does not have a remineralisation effect, it was necessary to add remineralisation agents to dental composites that contain MPC [29]. However, in this study, the group with MPC was confirmed to exhibit remineralisation capability and that this ability was improved when agents with remineralisation abilities were added. This can be inferred from the study of Lee et al., who added MPC to SPRG-filled RBC. They discovered that the release of ions increased with the amount of MPC increased, and that the synergistic effect was due to the properties of both ion release and acid neutralisation, which may be due to the structure of the MPC [32]. MPC can interact with liquid by arranging the polar head facing outward, with the non-polar tail facing inward, in the bilayer. Meanwhile, the negatively charged polar group has a catalytic effect on the surface [31]. Thus, we assume that the anti-demineralisation performance of MPC itself is due to the increased interaction with ions present in the remineralisation solution. Likewise, when MBN is added to MPC, the increased catalytic effect due to the addition of MPC increases the exchange rate of the calcium and phosphate ions of BAG, thus increasing the anti-demineralisation performance. The fact that the anti-demineralisation effect increased with the amount of MBN added can be interpreted through the same perspective.

In this study, the mechanical properties and protein adsorption, antibacterial, and anti-demineralisation abilities of orthodontic bonding agents with MPC and MBN were investigated. MPC supposedly improves the effect of agents with bactericidal effect, and thus we added MBN to MPC to investigate the antibacterial effect. However, in this study, the antibacterial effect of MPC itself was significant, such that the bactericidal effect of the added MBN was not significantly increased. Further studies on the bactericidal effect are necessary. In addition, we looked at the anti-demineralisation performance of the synthesised orthodontic bonding agents via the pH-cycling method. Future studies on pH measurement, to confirm if these orthodontic bonding agents neutralise the pH of the biofilm, and on long-term stability, to determine whether the pH is maintained for a long time, are necessary.

## 5. Conclusions

Orthodontic bonding agents with MPC and MBN have clinically acceptable mechanical properties and biological stability. As reported in previous studies, 5% MPC led to gelation, which reduced the mechanical properties and protein-repellent abilities of the bonding agents. MPC itself has protein-repellent and antibacterial effects, but these properties improved with the addition of MBN. Likewise, the anti-demineralisation effect of MBN itself increased with the addition of MPC. This study confirmed that the addition of MBN, which has anti-demineralisation ability, to MPC, which has protein-repellent and antibacterial abilities, improved all the aforementioned abilities, and demonstrated that this combination can be an option for preventing WSL. Furthermore, it can be applied to fields such as medical devices or bone regeneration that require HAp formation and antibacterial environment.

## Figures and Tables

**Table 1:** Tested groups.

Groups	CharmFil Flow (wt%)	MPC (wt%)	MBN (wt%)
CF (Control)	100%	0%	0%
3% MPC	97%	3%	0%
5% MPC	95%	5%	0%
3% MPC + 3% MBN	94%	3%	3%
3% MPC + 5% MBN	92%	3%	5%

**Table 2:** Adhesive remnant index (ARI) score and criterion.

Score	Criterion
1	All the adhesive remained on the tooth
2	More than 90% of the adhesive remained on the tooth
3	Between 10–90% of the adhesive remained on the tooth
4	Less than 10% of the adhesive remained on the tooth
5	No adhesive remained on the tooth

**Table 3:** Adhesive remnant index (ARI) scores of tested orthodontic bonding agents.

ARI	1	2	3	4	5	Sig.
CF	0	3	5	2	0	Not significant
3% MPC	0	2	4	4	0	
5% MPC	0	0	3	6	1	
3% MPC + 3% MBN	0	1	5	4	0	
3% MPC + 5% MBN	0	1	3	5	1	

* ARI scores were not significantly different, according to Kruskal–Wallis test at α = 0.05 (*n* = 10)

## Supplementary Materials

The following are available online at https://www.mdpi.com/2079-4991/10/7/1282/s1, Figure S1: Flow chart of mesoporous bioactive glass nanoparticle (MBN) synthesis, Table S1: Components of CharmFil Flow, Table S2: Compositions of demineralising and remineralising solutions.
