# Supplementary material for: Synergetic Effect of 2-Methacryloyloxyethyl Phosphorylcholine and Mesoporous Bioactive Glass Nanoparticles on Antibacterial and Anti-Demineralisation Properties in Orthodontic Bonding Agents
Source: Nanomaterials (Basel). 2020 Jun 30;10(7):1282. doi: 10.3390/nano10071282 (PMC7407988; doi:10.3390/nano10071282)
Supplement: Supplementary file 1 [file nanomaterials-10-01282-s001.pdf]

Appendix A

Distilled water

← Ethanol
Aqueous ammonia
2-ethoxyethanol
Calcium nitrate tetrahydrate
CTAB

Stirring for 30 min at room temperature

← TEOS

Stirring for 30 min at room temperature

← TEP

Stirring for 4 h at room temperature

Drying for 24 h at 60 °C

Calcination for 5 h at 600 °C

Sieving

Figure S1. Flow chart of mesoporous bioactive glass nanoparticle (MBN) synthesis.

CTAB, cetyl trimethyl ammonium bromide; TEOS, tetraethyl orthosilicate; TEP, triethyl phosphate.

Table S1. Components of CharmFil Flow.

Composition
Bis-GMA
Barium glass (Range of particle size: 0.7 μm)
Triethyleneglycol dimethacrylate
Diurethane dimethacrylate
Others

Table S2. Compositions of demineralising and remineralising solutions.

Demineralising solution (pH 4.4)		Remineralising solution (pH 7.0)	
Calcium 2.0 mmol/L	$Ca(NO_3)_2 \cdot 4H_2O$	Calcium 1.5 mmol/L	$Ca(NO_3)_2 \cdot 4H_2O$
Phosphate 2.0 mmol/L	KH_2PO_4	Phosphate 0.9 mmol/L	KH_2PO_4
Acetic acid 75.0 mmol/L	CH_3COOH	KCl 130.0 mmol/L	KCl
		Sodium cacodylate 20.2 mmol/L	$NaC_2H_6AsO_2 \cdot 3H_2O$
